# Epidemiological description of a protracted cholera outbreak in Tonj East and Tonj North counties, former Warrap State, South Sudan, May-Oct 2017

**DOI:** 10.1186/s12879-018-3640-5

**Published:** 2019-01-03

**Authors:** Fred Nsubuga, Stephen Chol Garang, Mathew Tut, David Oguttu, Robert Lubajo, Dennis Lodiongo, Michael Lasuba, Allan Mpairwe

**Affiliations:** 1 World Health Organization Warrap Hub, Juba, South Sudan; 2 Ministry of Health, Ministry Complex, Juba, South Sudan; 3 Ministry of Health, Kampala, Uganda; 4 Ministry of Health, Public Health Laboratory, Juba, South Sudan; 5 World Health Organization Country Office, Juba, South Sudan

**Keywords:** Cholera, Outbreak, Oral cholera vaccination, South Sudan

## Abstract

**Background:**

On 18th May 2017, State Ministry of Health of former Warrap State received a report from Tonj East County of an outbreak of acute watery diarrhoea and vomiting in Makuac payam. We conducted this investigation to confirm the causative organism and describe the epidemiology of the outbreak in order to support evidence-based control measures.

**Methods:**

We defined a suspected case as a resident of Tonj East or Tonj North County with sudden onset of acute watery diarrhoea and vomiting between May 1 and October 15, 2017. A probable case was defined as a suspected case with a positive rapid test for *Vibrio cholerae*; a confirmed case was a probable case with a positive stool culture for *V*. *cholerae*. We conducted systematic case finding by visiting health facilities and villages in the affected payams. We reviewed patient records from 1 May 2017 to 15 October 2017, to identify suspected cholera case-patients. We conducted a descriptive epidemiologic study, examining the distribution of the cases. We computed the attack rates by age, sex, and payam of residence. Case fatality rate was calculated as the ratio of the total number of suspected cholera death to the total number of cholera case-patients. We conducted an oral cholera vaccination campaign after the peak of the outbreak to control and prevent the spread to other payams.

**Results:**

We identified 1451 suspected cholera cases between May and October 2017. Of these, 81% (21/26) had a positive rapid diagnostic test for *V. cholerae*; out of the 16 rectal swabs transported to the National Public Laboratory, 88% (14/16) were confirmed to be *V. cholerae* O1 serotype Inaba. The epidemic curve shows continuous common source outbreak with several peaks. The mean age of the case-patients was 24 years (Range: 0.2-75y). The clinical presentations of the case-patients were consistent with cholera. Males had an attack rate of 9.9/10000. The highest attack rate was in ≥30y (14 per 10,000). Among the six payams affected, Makuac had the highest attack rate of 3/100. The case fatality rate (CFR) was 3.0% (44/1451). Paliang and Wunlit had an oral cholera vaccination coverage of ≥100%, while 4 payams had a vaccination coverage of < 90%.

**Conclusion:**

This was a continuous common source cholera outbreak caused by *V. cholerae* 01 sero type Inaba. We recommended strengthening of the surveillance system to improve early detection and effective response.

## Background

Cholera is a diarrheal disease caused by *V. cholerae* [1, 2]. It remains an important global health problem with several hundreds of thousands of reported cases each year. Despite all the major advances in research, the condition still remains a challenge to the modern medical world [3].

About 2.8 million cholera cases occur annually in endemic countries, among whom an estimated 91,000 die. In non-endemic countries, an estimated 87,000 cholera cases occur among whom 25,000 die. The incidence is estimated to be greatest in children less than 5 years of age [4].

Sub-Saharan Africa has the highest burden of cholera cases globally. Though there has been a decrease in the endemicity and intensity of epidemics across the continent, the case fatality rates remain higher in Africa than elsewhere [5]. Some of the risk factors that contribute to these outbreaks include; water contamination, heavy rainfall and flooding and population displacement [6]. It has also been associated with poverty and closely linked to inadequate drinking water and poor sanitation. In South Sudan, a cholera outbreak occurred in Juba between January and June 2007, with around 3157 suspected cholera cases and 74 death [7]. In 2014, another cholera outbreak occurred in Juba county, South Sudan during a major humanitarian crisis that was triggered by political and ethnic tension [8].

On 18th-May-2017, former Warrap State Ministry of Health received a report from Tonj East County of an outbreak of acute watery diarrhoea and vomiting in Makuac payam. Two adult patients had reported to Makuac Primary Health Care Unit (PHCU) on the 17th-05-2017, with severe dehydration and were referred to Maria Lou Hospital for further management. On 18th-May-2017, two case-patients with similar presentation were admitted in Makuac PHCU from Wuncuei and Paliang PHCU. Preliminary assessment by the County Health Department (CHD) manager and county surveillance officer showed that these case-patients developed these symptoms a few hours after eating food. The report also highlighted that there were 8 suspected acute watery diarrhoea (AWD) deaths, with 22 AWD admissions in the different health facilities. Initial testing with cholera rapid diagnostic test showed the samples were positive. We conducted this investigation to confirm the causative organism and describe the epidemiology of the outbreak in order to support evidence-based control measures.

## Methods

### Study site

**Fig. 1:**
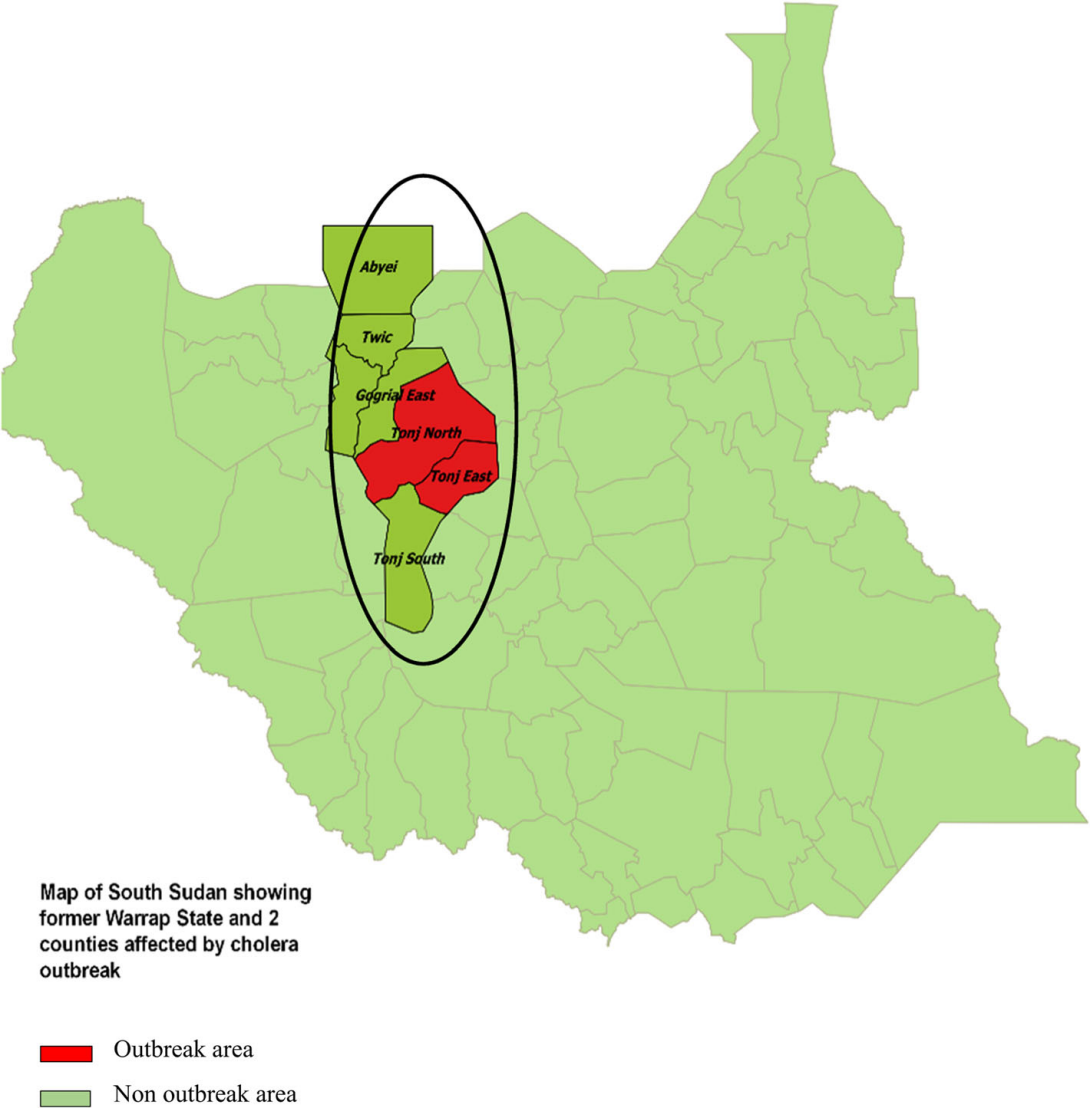
Map of South Sudan showing former Warrap State and Tonj East and Tonj North Counties affected by cholera outbreak May-Oct 2017

The outbreak occurred in Tonj East and Tonj North Counties, in the former Warrap State in the Republic of South Sudan (Fig. 1). These two counties are composed of 16 payams of which 6 were affected. A payam is the second-lowest administrative division, below a county. It has a minimum population of 25,000. Tonj East had 5 affected payams namely: Makuac, Paweng, Wunlit, Palal and Paliang; while Maria-Lou was the only affected payam from Tonj North County. The projected population of the area is 412,969 of whom 16,519 (4%) are children ≤1 year. The main economic activity in the area is cattle keeping.

### Case definition

A suspected case was a resident of Tonj East or Tonj North County with sudden onset of acute watery diarrhoea and vomiting between May and October, 2017. A probable case was a suspected case with a positive rapid test for *V. cholerae*; a confirmed case was a probable case with a positive stool culture for *V. cholerae*. A cholera death was death of a suspected, probable or confirmed cholera case-patient.

### Case finding

We conducted systematic case finding by visiting health facilities and villages in the affected payams. We reviewed patient records from 1 May 2017 to 15 October 2017 to identify suspected cholera case-patients. We recruited and trained health workers, surveillance officers, community health workers and village chiefs on case identification using the case definition. We visited the case-patients’ homes and other health facilities to verify the cases. We used case investigation questionnaires to obtain demographic and epidemiological information for descriptive analysis. Those meeting the case definition were referred to cholera treatment units (CTU) for further management. The community health workers were also responsible for referral of patients from the villages to CTUs.

### Descriptive epidemiologic analysis

We conducted a descriptive epidemiologic study, examining the distribution of the cases. We constructed an epidemic curve to examine the development of the epidemic over time.

We described the main clinical symptoms and signs of the case-patients. We computed the attack rates by age, sex, and payam of residence. Case fatality rate was calculated as the ratio of the total number of suspected cholera death to the total number of case-patients. The administrative coverage for OCV was estimated by dividing the number of people who received the vaccine by all residents >1y in a particular payam using 2008 census projected figures. This was used because the National Bureau of Statistics for South Sudan, usually projects population figures based on population growth rates to support planners at both state and national level [9].

### Laboratory analysis

In the field, stool specimens were collected before patients received antimicrobial treatment and tested using Crystal VC™ rapid diagnostic test (Span Diagnostics, Surat, India). The results were read within 10–15 min and interpreted following the manufacturer’s recommendation. The test was considered positive if the control line and either T2(O1) or T1 (O139) or both (O1 and O139) appeared; negative if the control line only appeared and invalid if the control line did not appear [10, 11]. The reported sensitivity for Crystal VC™ RDT under field conditions was 93.4 (95% CI: 88.7–96.2), and specificity was 49.2 (95% CI: 44.3–54.1) [12]. RDT reactive stool specimens were transported in Cary-Blair media at 2-8 °C to Public Health Laboratory (PHL) Juba for bacteriological analysis.

### Stool culture and bacterial identification

Stool specimens were inoculated in alkaline peptone water, incubated at 35-37 °C for 4 h, plated on thiosulfate citrate bile salts sucrose (TCBS) agar plates and incubated overnight at 35–37 °C. Culture plates were visually examined for medium-sized convex, smooth, yellow colonies and sub-cultured on nutrient agar plates overnight at 35–37 °C. Colonies from the nutrient agar were screened using oxidase discs (Himedia Laboratories Pvt. ltd, Mumbai, India) and oxidase positive isolates serotyped using polyvalent O1 specific antiserum (Bio-Rad, USA) and monovalent Inaba and Ogawa antisera (Denka Seiken Co, Japan).

### Case management

CTU (Makuac, Paweng & Paliang) and 4 Oral rehydration points (ORPs) (ager bac, Aliet, Apiir nhom & Mapara) were set up to support in the isolation and management of cholera case-patients. All cholera case-patients were referred to these designated areas for further management

### Oral cholera vaccination with Shanchol

Due to fear of escalation of the outbreak to surrounding communities, South Sudan Ministry of Health (MOH), World Health Organization (WHO), International Organization for Migration (IOM) and other partners agreed to conduct an oral cholera vaccination in the risk communities and around the affected areas. However, because of limited vaccines and other logistical challenges the campaign was conducted in 7 payams out of the 16 payams in the affected counties. Among the targeted 7 payams, 6 had confirmed cholera patients and the seventh had limited health services with very high risk for spread. The State Ministry of Health quantified the required OCVs, human resources, and other logistics. A total of 189,058 OCV doses were delivered to support the first round of the vaccination that was conducted on the 6th Aug 2017.

## Results

**Fig. 2:**
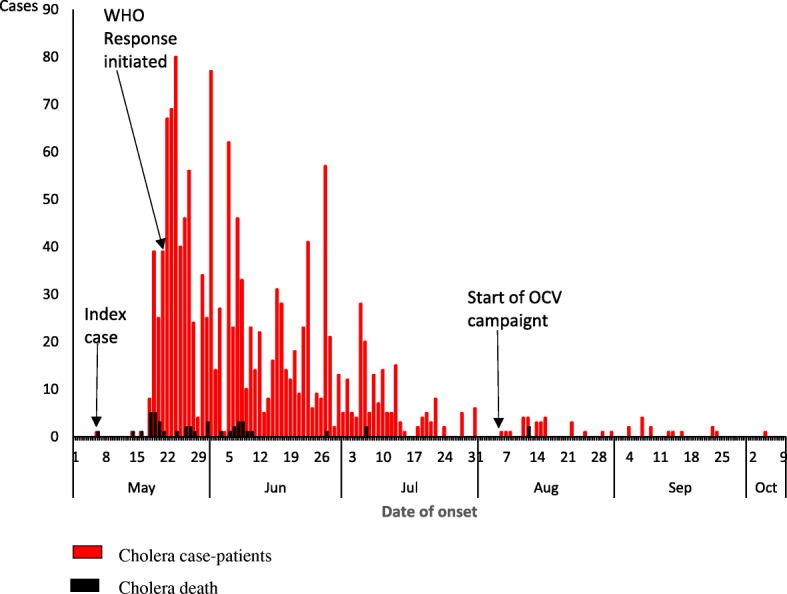
Epi curve showing number of cholera cases by onset date: Tonj East and Tonj North Counties, Warrap state, South Sudan May-Oct, 2017

**Fig. 3:**
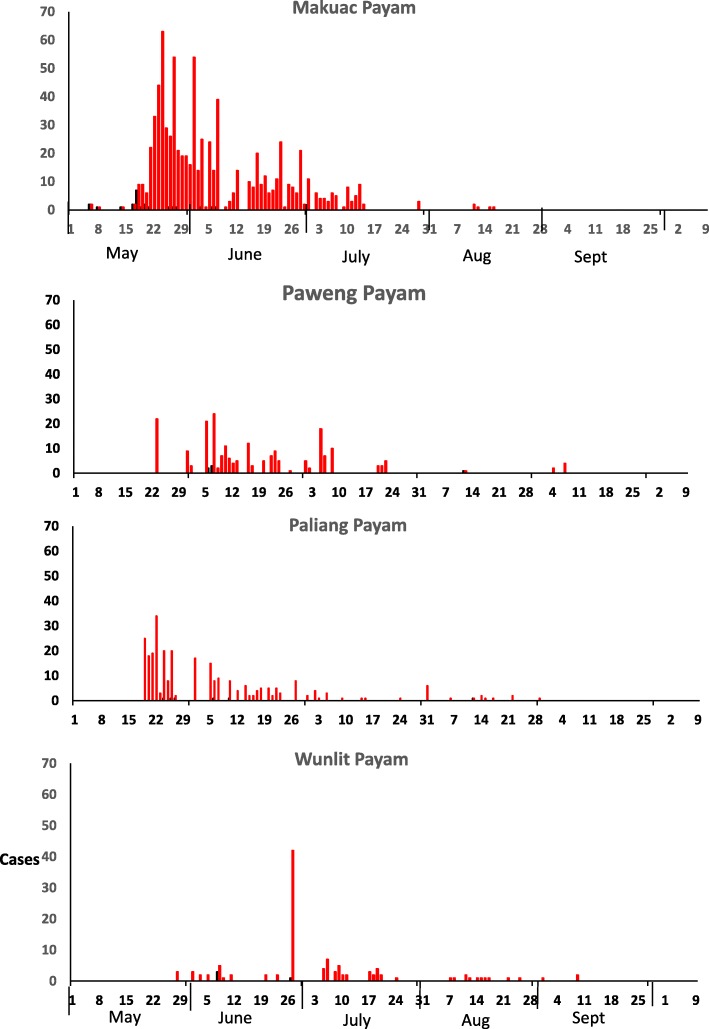
Epidemic curves of cholera cases in the 4 most affected payams: Tonj East and Tonj North Counties, Warrap state, South Sudan May-Oct, 2017

During the outbreak that occurred in Tonj East and Tonj North Counties between May and October 2017, 2% (26/1451) of the suspected cholera cases had stool samples collected and tested using RDTs. Of those tested at the different treatment centers, 81% (21/26) were positive with RDT. Out of the 16 stool samples transported to the PHL, 88% (14/16) were confirmed to be *V. cholerae* 01 serotype Inaba. The epidemic curve shows continuous common source outbreak with several peaks (Fig. 2). The index case that succumbed on 6th-05-2017, showed symptoms of cholera as he was returning from a cattle camp near former Unity State which shares a border with the Republic of Sudan. Due to poor infrastructure, inaccessible roads, insecurity, lack of telephone communication network and inadequate knowledge about the illness, most of the initial cases died as they returned from the cattle camps to their communities. The time lag between the index case and notification was due to surveillance challenges. The highest number of cases had onset on 24th May, 2017 in epidemiological week 20, while the country-wide epidemic peak occurred in epidemiological week 24. The distribution of the cases by time of onset in the four most affected payams has also been shown (Fig. 3). The case fatality rate (CFR) was 3.0% (44/1451). The oral cholera vaccination campaign was started on 6th August, 2017 and lasted for 5 days. The mean age of the case-patients was 24 years (Range: 0.2-75y). The clinical presentations of the case-patients were consistent with cholera (Table 1).

**Table 1:** Clinical presentation of cholera case-patients: Tonj East and Tonj North Counties, former Warrap state, South Sudan May–October, 2017

Clinical features	% *n* = 1451
Acute watery diarrhoea	100
Vomiting	100
Dehydration	99

Males and females had similar attack rates. The highest attack rate was in ≥30y (14 per 10,000) (Table 2).

**Table 2:** Attack rate of cholera per 10,000 during an outbreak in Tonj East and Tonj North Counties, former Warrap state, South Sudan, May to October, 2017

Characteristic	Frequency	Population	Attack rate/10000
Sex			
Female	722	783,373	9.2
Male	728	732,125	9.9
Age-group			
0–0.9	28	60,620	4.6
1–5	182	257,635	7.1
6–15	334	424,339	7.9
16–29	377	394,029	9.6
≥ 30	530	378,875	14

Among the six payams, Makuac had the highest attack rate (Table 3).

**Table 3:** Attack rate of cholera by payam: Tonj East and Tonj North Counties, former Warrap state, South Sudan May–October, 2017

Payam	Frequency	Population	Attack rates/100
Makuac	800	26,830	3.0
Paliang	281	23,820	1.2
Paweng	216	26,962	0.8
Palal	3	17,067	0.02
Maria Lou	31	24,476	0.1
Wunlit	113	35,721	0.3
Total	1444	154,876	0.9

Paliang and Wunlit had an oral cholera vaccination coverage of ≥100%, while 4 payams had a vaccination coverage of < 90% (Table 4).

**Table 4:** 1st round coverage of oral cholera vaccination by payam: Tonj East and Tonj North Counties, former Warrap state, South Sudan May-Oct, 2017

Payam	Target	# Vaccinated	% Coverage
Ananatak	38,400	25,135	65
Makuac	25,757	20,945	81
Palal	16,384	12,600	77
Paliang	22,867	28,110	123
Paweng	25,884	25,405	98
Wunlit	34,292	34,650	101
Maria-Lou	23,497	14,017	60
Total	187,081	160,862	86

## Discussion

Our investigation revealed that a continuous common source outbreak of *V. cholerae* 01 serotype Inaba occurred in Tonj East and Tonj North Counties, in the Republic of South Sudan, with a CFR of 3.0%. The highest attack rate was in age ≥ 30y; there was no difference in gender. The administrative coverage for Shanchol oral cholera vaccination was < 85% in 4(57%) of the payams.

*V. cholerae* 01, serotype Inaba has been known to cause outbreaks with high morbidity and mortality [13]. Epidemics occur after war, civil unrest, or natural disasters when water and food supplies become contaminated with *V. cholerae* in areas with crowded living conditions and poor sanitation. Its ability to cause death within hours of onset especially in undeveloped countries has become a major public health challenge. It is therefore important that awareness about cholera outbreaks is created to stimulate better understanding of the disease and lead to development of practical preventive and therapeutic measures [14]. Death of cholera case-patients usually results from profuse secretory diarrhoea which leads to severe dehydration, metabolic acidosis, electrolyte imbalance and circulatory collapse [15]. A study conducted in Juba city, South Sudan confirmed *V. cholerae* 01 as the cause of the outbreak with over 6000 cases [16]. Studies done elsewhere have showed *V. cholerae* as a cause of wide spread outbreaks [17, 18].

The continuous common source outbreak that occurred in Tonj, between May and October 2017 was attributed by several factors; these included the inadequate surveillance system and insecurity that hampered the movement of the investigation teams from doing a thorough environmental assessment. The inability of the team to effectively identify the source of the outbreak prolonged the exposure beyond one incubation period [19]. Several studies have demonstrated that cholera outbreaks can persist in the community if not adequately investigated [7, 20].

The CFR has been known to be a measure of the adequacy of the health care system in cholera outbreak response [21]. Our investigation showed the CFR as 3.0%, a figure that is more than two fold higher than the recommended WHO standard of ≤1%. Such high CFR suggests a failure in preparedness, surveillance, case management, poor provision of water, inadequate sanitation and response [22]. An assessment done in the 2010 cholera outbreak response in northern Nigeria showed a CFR of 3.75% [22], higher than what was recorded in our study. Another study done in South Sudan showed a CFR as high as 11% in some counties [16]. Studies done elsewhere have showed CFRs lower than what was recorded in our study [23].

Our study found the highest attack rate to be among age ≥ 30y, with no significant difference in gender. This could have been due to the fact that this age group was involved in the care and transportation of the cholera case-patients from the villages to the CTUs with subsequent contamination. This contrasts a study done in Kasese District, Western Uganda which showed the highest attack rate among 5-14y at 4.2%, though there was also no difference in gender. The WHO position paper highlights that young children living in endemic areas are the most affected by the disease but any age group may suffer [24].

Oral cholera vaccines (OCVs) have been recommended in cholera-endemic settings and pre-emptively during outbreaks and complex emergencies [25]. This particular OCV was conducted to prevent the spread of the outbreak to other areas. This notwithstanding, there were gross challenges in the mobilization of vaccines, logistics and human resources to undertake this campaign. This accounted for the delay in the implementation of this campaign. The overall coverage achieved during this OCV campaign in response to this outbreak was 86%. This is of major public health importance because OCVs have been shown to confer herd immunity in areas where 50% vaccination coverage has been achieved [24]. This cholera outbreak was controlled within a period of one month following implementation of the oral cholera vaccination.

### Strength and limitations

This study reveals the challenges and gaps experienced in countries with insecurity regarding, prevention, early detection and effective response to public health threats. However, it also has limitations: Owing to surveillance challenges and insecurity in the area, several cases could have been missed at the beginning of the outbreak, which limits the reliability of our descriptive epidemiology. Also, due to tribal wars and conflicts in most counties that surrounded Tonj, there were massive population movements from areas which were affected by the insurgency to areas that were relatively calm. We were unable to adjust for the population movements during this study which may have affected our denominator leading to coverages above 100%.

## Conclusions

This was a continuous common source cholera outbreak caused by Vibrio cholerae serotype Inaba. We recommended strengthening of surveillance system to improve detection and response.

## Abbreviations

CHD: Community health department
CTU: Cholera treatment unit
OCV: Oral cholera vaccine
PHCU: Primary health care unit
UNICEF: United nations children fund
WHO: World health organization
